# Unc5B Interacts with FLRT3 and Rnd1 to Modulate Cell Adhesion in *Xenopus* Embryos

**DOI:** 10.1371/journal.pone.0005742

**Published:** 2009-05-29

**Authors:** Emil Karaulanov, Ralph T. Böttcher, Peter Stannek, Wei Wu, Marlene Rau, Souichi Ogata, Ken W. Y. Cho, Christof Niehrs

**Affiliations:** 1 Division of Molecular Embryology, DKFZ-ZMBH Alliance, German Cancer Research Center, Heidelberg, Germany; 2 Department of Developmental and Cell Biology, Developmental Biology Center, University of California Irvine, Irvine, California, United States of America; The University of Hong Kong, China

## Abstract

The FLRT family of transmembrane proteins has been implicated in the regulation of FGF signalling, neurite outgrowth, homotypic cell sorting and cadherin-mediated adhesion. In an expression screen we identified the Netrin receptors Unc5B and Unc5D as high-affinity FLRT3 interactors. Upon overexpression, Unc5B phenocopies FLRT3 and both proteins synergize in inducing cell deadhesion in *Xenopus* embryos. Morpholino knock-downs of Unc5B and FLRT3 synergistically affect *Xenopus* development and induce morphogenetic defects. The small GTPase Rnd1, which transmits FLRT3 deadhesion activity, physically and functionally interacts with Unc5B, and mediates its effect on cell adhesion. The results suggest that FLRT3, Unc5B and Rnd1 proteins interact to modulate cell adhesion in early *Xenopus* development.

## Introduction

FLRT proteins comprise a small family of fibronectin type III domain (FNIII) and leucine-rich repeats (LRR) transmembrane proteins in vertebrates [1]. FLRT3, the best-characterized member of the family, can physically interact with FGF receptors and modulate FGF-ERK signalling [2]. In addition, FLRT3 induces homotypic cell sorting in cultured cells and in *Xenopus* embryos [3]. FGF signalling is dependent on the cytoplasmic tail, whereas FGF receptor binding is mediated by the FNIII domain of FLRT3 [2]. The extracellular LRR domains are dispensable for FGF signal activation [2], but are essential for the FLRT3-mediated cell sorting activity [3]. FLRT3 was also identified as a target gene of Nodal signalling, inhibiting cadherin adhesion in *Xenopus* early development through interaction with the Rho family GTPase Rnd1 [4]. In the mouse, FLRT3 knockout embryos display defects in ventral closure, headfold fusion and definitive endoderm migration [5], as well as disorganization of the basement membrane which leads to rupture of the anterior visceral endoderm [6], suggesting that cell adhesion is affected upon FLRT3 ablation. Furthermore, FLRT3 has been implicated in neurite outgrowth [7], [8].

The Netrin receptor Unc5 of the immunoglobulin (Ig) superfamily is involved in axon guidance, vasculogenesis and apoptosis regulation. Unc5 was originally identified as a mutation in the nematode *C. elegans* resulting in an uncoordinated movement phenotype [9]. Four Unc5 paralogs (A to D) are present in vertebrates, where they regulate axon migration together with the Netrin coreceptor DCC [10]. In addition, Unc5 proteins show properties of dependence receptors and can induce apoptosis through their Death domain in the absence of Netrin binding [11], [12]. Unc5B also plays a role in vascular morphogenesis [13], [14]. Despite the important roles ascribed to the Unc5 receptors, currently there is limited knowledge of their binding partners, modulators and downstream targets.

Through a cell surface binding screen for FLRT3 partners, we identified Unc5B and Unc5D as high-affinity FLRT3 interactors. We show that FLRT3 and Unc5B functionally interact in modulating cell adhesion during early *Xenopus* development, and provide evidence that the Unc5B effect on adhesion is mediated by Rnd1.

## Results

### FLRT3 binds to Unc5

**Figure 1:**
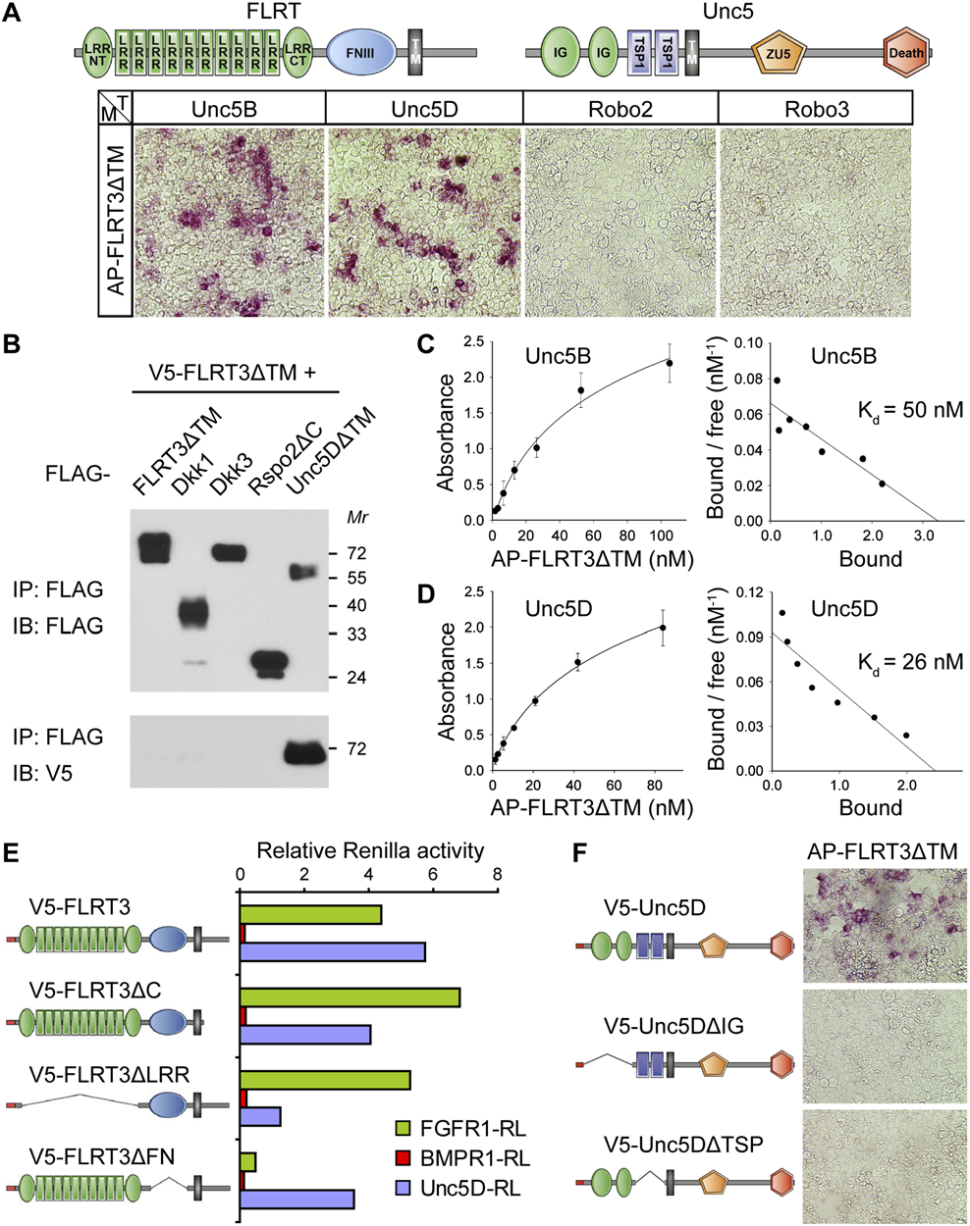
Unc5 proteins are high affinity interactors of FLRT3. (A) FLRT3 ectodomain binds to cells expressing Unc5. HEK293T cells were transfected (T) with the indicated expression constructs, incubated with AP-tagged FLRT3ΔTM conditioned medium (M) and stained for bound alkaline phosphatase (AP) activity. Schematic representation of the FLRT and Unc5 family domain structures is shown on top. (B) FLRT3 and Unc5D ectodomains interact in solution. Equal amounts of V5-tagged FLRT3ΔTM conditioned medium were mixed with the indicated FLAG-tagged condition media and subjected to immunoprecipitation (IP) and immunoblotting (IB). (C,D) Binding curves and Scatchard analyses of AP-FLRT3ΔTM binding to Unc5B or Unc5D transfected cells. The dissociation constants (K_d_) are indicated. (E) The LRR domains of FLRT3 mediate binding to Unc5D. Whole cell lysates of HEK293T cells transfected with the indicated Renilla- (RL-) and V5-tagged constructs were immunoprecipitated with anti-V5 antibody. RL activity of individual samples after IP was normalized to the total activity of the respective lysate. (F) Both the IG and the TSP domains of Unc5D are required for FLRT3 binding. HEK293T cells were transfected with the indicated V5-tagged Unc5D expression constructs, incubated with recombinant AP-FLRT3ΔTM and stained for bound AP activity. Similar levels of protein production were confirmed by V5 immunoblotting (data not shown).

To identify interacting partners of the *Xenopus* FLRT3 ectodomain, we performed a cell surface binding screen [15]. Pools of about 250 clones prepared from a mouse embryonic cDNA expression library were transfected in HEK293T cells. Two days later, the cells were incubated with soluble alkaline phosphatase-FLRT3 ectodomain fusion protein (AP-FLRT3ΔTM) and screened for FLRT3 interactors by a chromogenic assay for cell-surface bound AP activity. In this screen, a splice variant of the mouse *Unc5D* gene was isolated. FLRT3 was found to also interact with *Xenopus* Unc5B. FLRT3 interaction with Unc5B and -D is specific, as no binding was observed to other receptors of the Ig superfamily, such as Robo2 and -3 (Figure 1A). Additional *in vitro* binding and coimmunoprecipitation assays confirmed that FLRT3 and Unc5D ectodomains interact in solution with high specificity (Figure 1B). Next, the binding affinity of the FLRT3 ectodomain to Unc5 was determined in equilibrium binding experiments. The dissociation constant (K_d_) of AP-FLRT3ΔTM was 50 nM with Unc5B and 26 nM with Unc5D (Figure 1C,D). These values are comparable to the Netrin-Unc5 K_d_ [16]. We conclude that Unc5 and FLRT3 ectodomains interact specifically and with high affinity.

**Figure 2:**
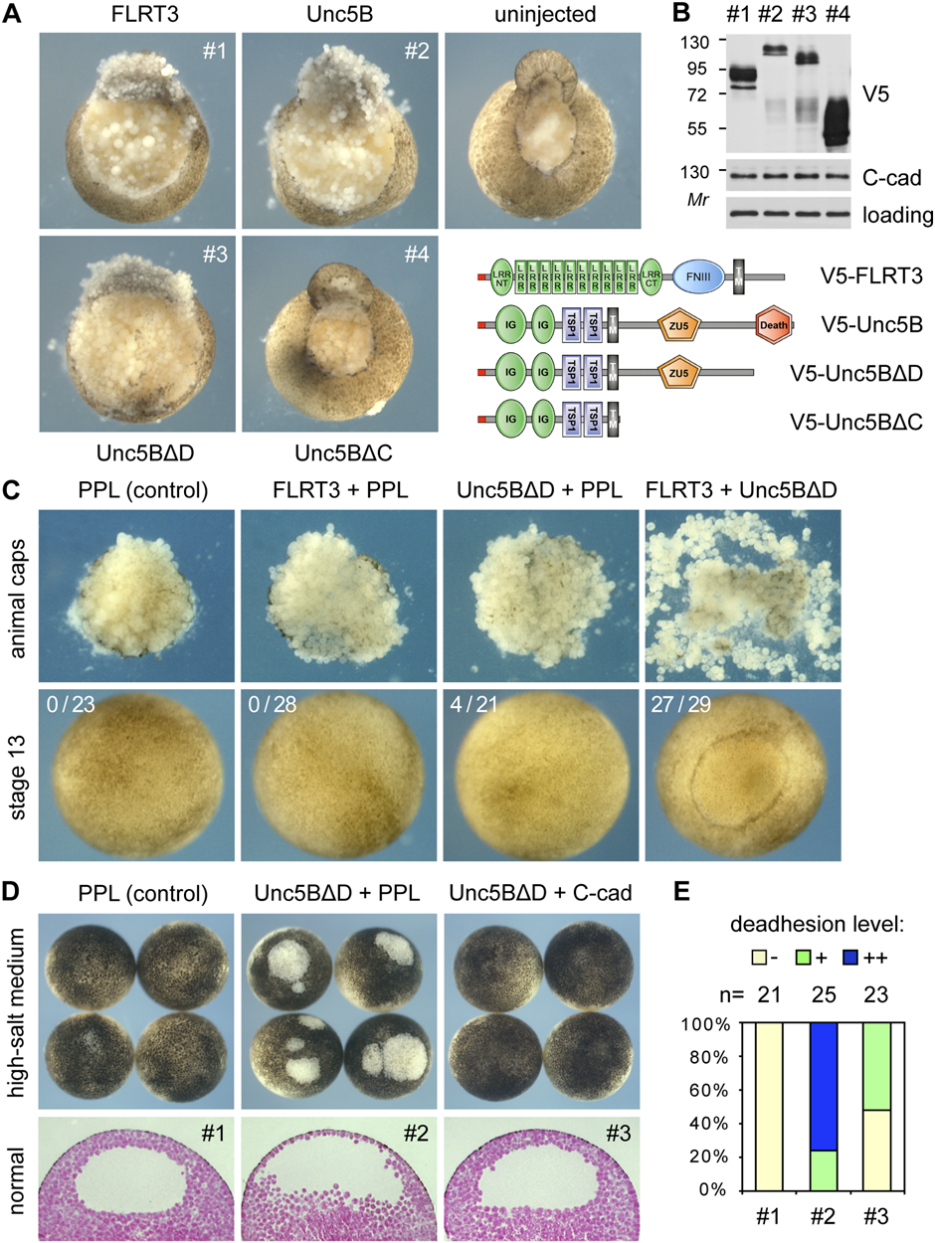
Unc5B and FLRT3 induce cell deadhesion in *Xenopus laevis* embryos. (A) Embryos were injected with the indicated mRNAs (1.2 ng FLRT3, 4 ng Unc5B variants) and at blastula stage 9 were photographed 10 min after partial excision of the animal caps. (B) V5 and C-cadherin immunoblots for protein production of sibling embryos from panel A. (C) FLRT3 and Unc5B functionally synergize. Embryos were injected with the indicated mRNAs (0.2 ng FLRT3, 1.6 ng Unc5BΔD) and cell deadhesion was assessed in animal caps of blastula stage 8.5 embryos (upper row), as well as in whole embryos at neurula stage 13, where ectodermal thinning and blastocoel edema were observed (lower row, indicated is the fraction of affected embryos). PPL, preprolactin (control mRNA). (D) C-cadherin rescue of Unc5B-induced deadhesion. Embryos were injected with the indicated mRNAs (3 ng Unc5BΔD, 1.5 ng C-cadherin) and grown until blastula stage 9 in high-salt medium, which promotes ectodermal lesions when adhesion is inhibited (upper row) or in normal medium (lower row, paraffin sections). (E) Quantification of the C-cadherin rescue effect in embryos from panel D (normal medium).

To further characterize the FLRT3-Unc5 interaction, a series of FLRT3 deletion constructs was used in coimmunoprecipitation experiments with Unc5D-Renilla fusion protein to determine the FLRT3 domain required for Unc5 binding. The interaction depends mostly on the LRR region of FLRT3 (Figure 1E), which is in contrast to the binding of FLRT3 to FGF receptors for which the FNIII domain is essential (Figure 1E and [2]). On the other hand, deletion of either the Ig- or the TSP-domains of Unc5D abolished FLRT3 interaction in cell surface binding experiments (Figure 1F), revealing that both domains are required for efficient FLRT3 binding.

### Unc5B and FLRT3 induce embryonic cell deadhesion

To analyse if FLRT3 and Unc5 proteins interact not only biochemically but also functionally, we carried out overexpression experiments in *Xenopus laevis* embryos, where mRNA injection of FLRT3 has been shown to induce cell deadhesion [4]. Similar to FLRT3, Unc5B mRNA injection induced loss of cell adhesion and inhibited wound healing (Figure 2A). Deletion of the intracellular part of Unc5B abolished the deadhesion phenotype (Figure 2A, Unc5BΔC), despite the fact that this construct was expressed at higher protein levels (Figure 2B). Expression of C-cadherin, which is the major cadherin in early *Xenopus* development [17] was not affected by Unc5B, as was also the case for FLRT3 (Figure 2B and data not shown). Unc5B effect on adhesion was specific and not due to cell toxicity or apoptosis, since (i) the detached cells remained viable and proliferative when cultured *ex vivo* (data not shown), (ii) deletion of the pro-apoptotic Death domain did not abolish cell deadhesion (Figure 2A, Unc5BΔD) and (iii) the phenotype could be rescued by C-cadherin overexpression or by Rnd1 depletion (see below). Nevertheless, to avoid potential toxic effects associated with the Death domain [10], subsequent experiments were performed with Unc5BΔD. We conclude that Unc5B phenocopies FLRT3 in inducing cell deadhesion and that this effect requires its cytoplasmic part (excluding the Death domain).

To test for their functional interaction, limiting mRNA doses of FLRT3 and Unc5BΔD were injected, which individually yielded no significant deadhesion (Figure 2C). Upon co-injection, however, a synergistic cell dissociation effect was observed in isolated animal caps and in whole embryos, indicating that both proteins can act together to interfere with cell adhesion (Figure 2C).

FLRT3-induced deadhesion was proposed to involve C-cadherin endocytosis and could be rescued by C-cadherin overexpression [4]. We therefore tested whether Unc5B operates through a similar mechanism. Indeed, C-cadherin was able to substantially rescue Unc5BΔD-induced deadhesion (Figure 2D,E), suggesting that Unc5B, like FLRT3, can interfere with cadherin-mediated cell adhesion in *Xenopus* embryos. However, we failed to observe significant C-cadherin internalization upon either Unc5BΔD or FLRT3 overexpression (data not shown).

### Unc5B-FLRT3 interaction in *Xenopus* morphogenesis

**Figure 3:**
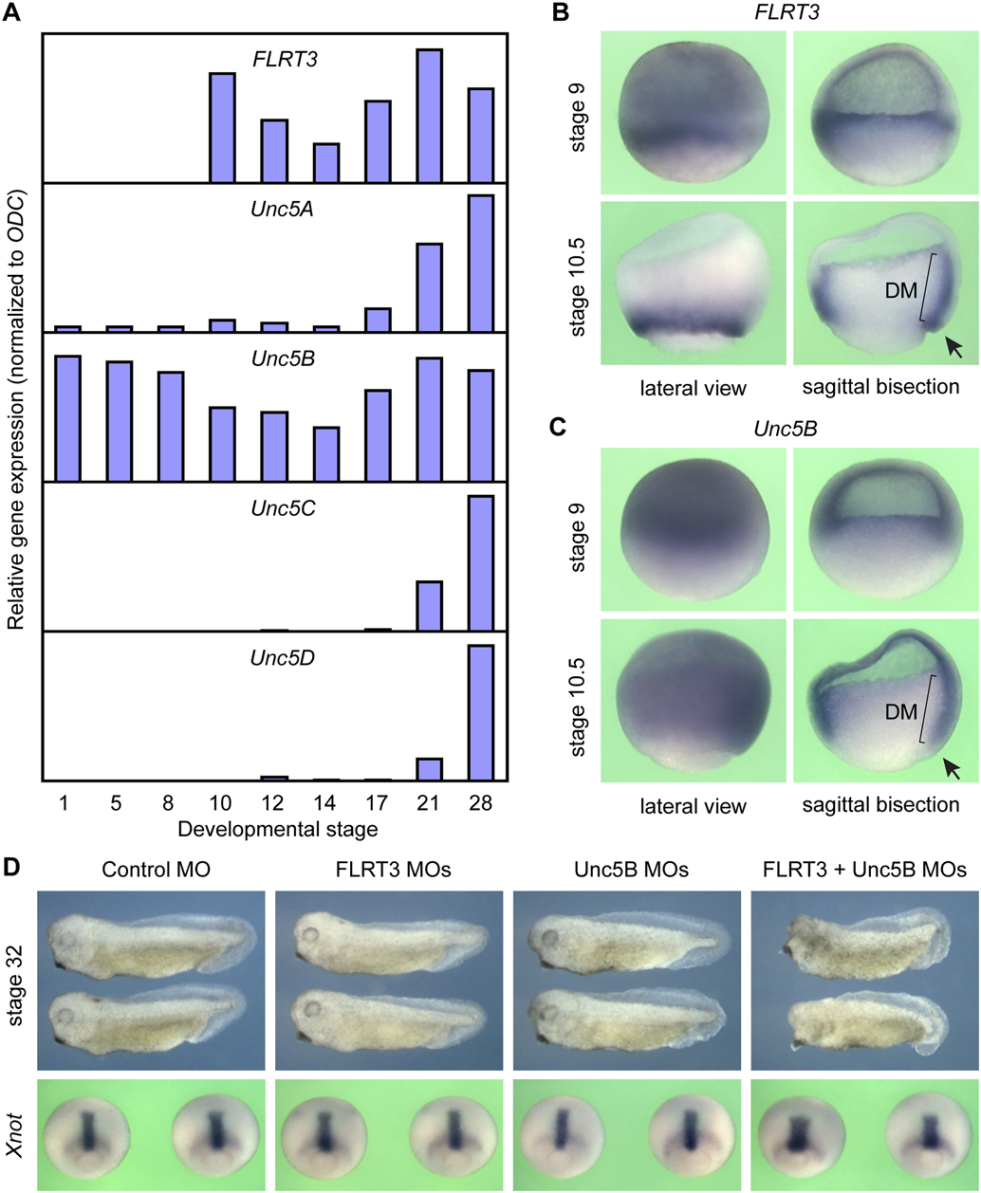
Unc5B-FLRT3 interaction in *Xenopus tropicalis* morphogenesis. (A) Developmental quantitative RT-PCR analysis of *FLRT3* and *Unc5A-D* expression. (B–C) Expression patterns of *FLRT3* and *Unc5B* at blastula stage 9 and gastrula stage 10.5. The dorsal blastopore lip is indicated with an arrow. DM, dorsal mesoderm. (D) Representative phenotypes of embryos injected with FLRT3 and/or Unc5B Morpholinos (MOs). A mixture of two MOs per gene targeting the ATG and the UTR regions was injected (10 ng each). A stunted axis was observed in 0% (n = 29), 3% (n = 34), 7% (n = 28) and 100% (n = 31) of the injected embryos, respectively. Lower row: whole mount *in situ* hybridization of *Xnot* at late gastrula stage 12.

Since multiple FLRT and Unc5 paralogs are expressed in early *X. laevis* development ([2],[4] and data not shown), and because of the existence of pseudoalleles in this tetraploid species, we chose the related diploid species *X. tropicalis* for loss-of-function experiments. Of the four *Unc5* paralogs, *Unc5B* proved the most abundantly expressed gene throughout *X. tropicalis* development (Figure 3A and data not shown). Comparison of the spatio-temporal expression patterns of *Unc5B* and *FLRT3* reveals overlapping expression domains in the mesoderm and the sensorial ectoderm at blastula stage, and later in the mesoderm at gastrula stage, tissues which undergo intense morphogenetic movements during gastrulation (Figure 3B,C). Also in later development the two genes show partially overlapping expression in certain tissues (Figure S1).

**Figure 4:**
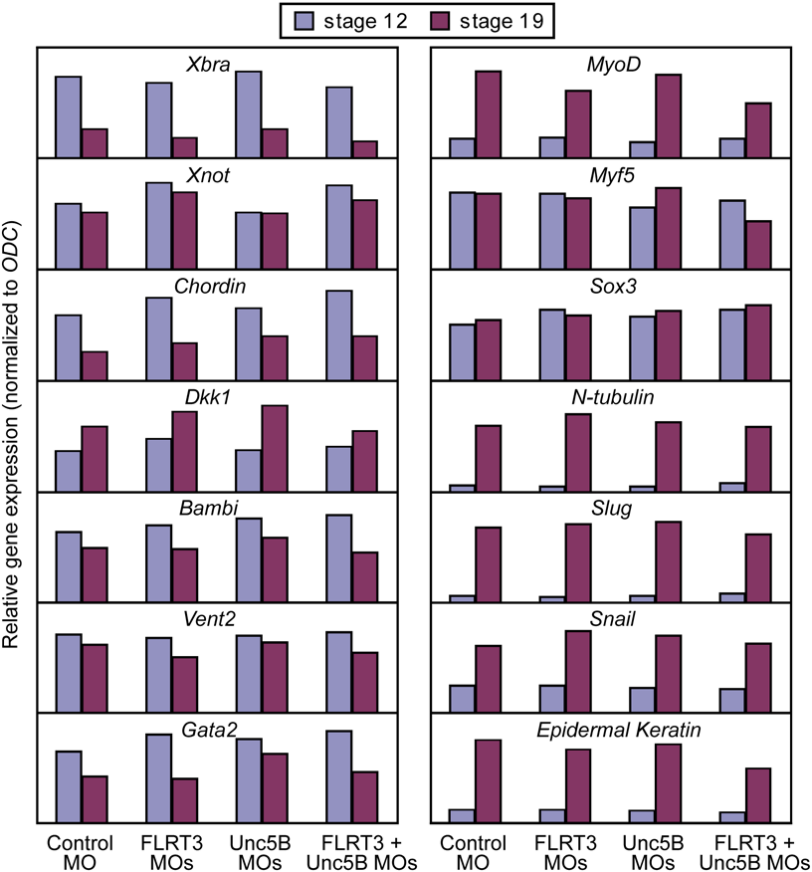
Marker gene expression analysis of the FLRT3/Unc5B morphant embryos. Stage 12 (late gastrula) and stage 19 (late neurula) sibling embryos of those analysed in Figure 3D were assessed by quantitative RT-PCR for expression of the tissue markers *Xbra* (mesoderm), *Xnot*, *Chordin* and *Dkk1* (dorsal mesoderm), *Bambi*, *Vent2* and *Gata2* (ventral mesoderm), *MyoD* and *Myf5* (paraxial mesoderm and somites), *Sox3* and *N-tubulin* (neuroectoderm and neurons), *Slug* and *Snail* (neural crest), and *Epidermal Keratin*.

In order to test for functional interaction, we injected *X. tropicalis* embryos with Morpholino (MO) antisense oligos targeting FLRT3 and Unc5B individually or in combination (Figure 3D). Because of a lack of suitable antibodies to assess endogenous protein depletion and in order to achieve efficient knock-down effects [18], we used a mixture of two non-overlapping MOs for FLRT3 and Unc5B, targeting their ATG and UTR regions. While individual injection of FLRT3 or Unc5B MOs did not significantly affect early development, combined FLRT3/Unc5B morphant embryos had shorter bodies and smaller heads, suggesting morphogenetic defects (Figure 3D). The expression domain of the dorsal mesodermal marker *Xnot* in gastrula stage embryos was shorter and broader, a hallmark of impaired convergent extension movements during gastrulation (Figure 3D). Quantitative RT-PCR analysis at gastrula and neurula stages revealed no major changes in the expression of epidermal, neuroectodermal, neural crest, paraxial, dorsal and ventral mesodermal marker genes (Figure 4). This indicates that cell fate determination and tissue differentiation were not significantly affected, consistent with morphogenetic rather than differentiation defects. The fact that (i) only combined FLRT3/Unc5B MO injection yields phenotypic defects, which (ii) concern the dorsal mesoderm where both genes are coexpressed, and (iii) leaves cell differentiation markers largely unaffected, supports the specificity of the Morpholino knockdowns. We conclude that Unc5B and FLRT3 functionally interact during early *Xenopus* development.

### Unc5B acts upstream of the small GTPase Rnd1

Both Unc5B and FLRT3, while interacting via their ectodomains, modulate cell adhesion through their cytoplasmic tails. The small GTPase Rnd1 was shown to bind to FLRT3 and to mediate its effect on cell adhesion [4]. We therefore tested whether Rnd1 can also interact with Unc5B.

**Figure 5:**
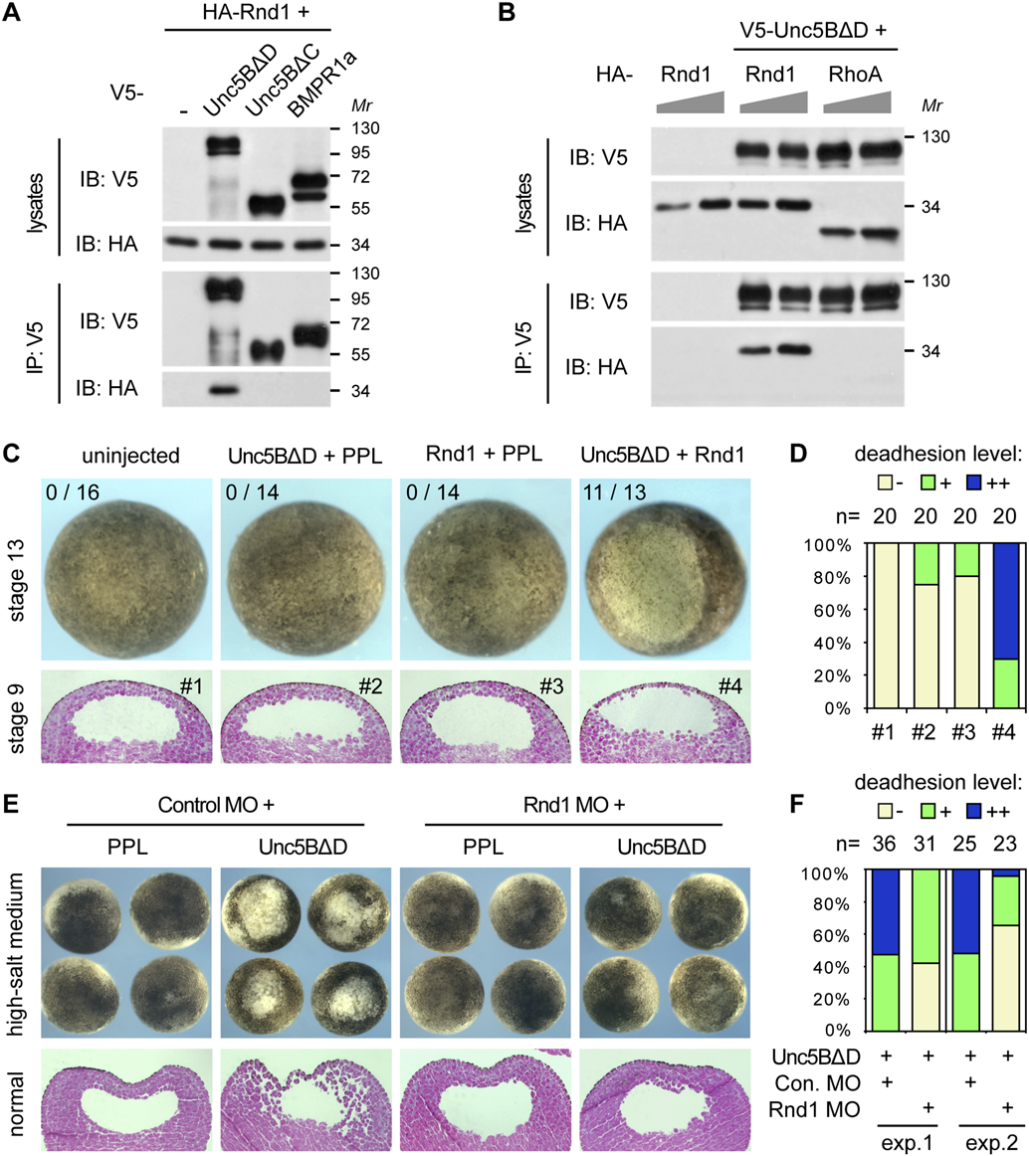
Unc5B acts upstream of the small GTPase Rnd1. (A,B) Rnd1 binds to Unc5BΔD. Immunoprecipitation (IP) assays followed by immunoblotting (IB) with lysates of HEK293T cells overexpressing the indicated V5- and HA-tagged proteins. (C) Unc5B and Rnd1 synergize in cell deadhesion. *X. laevis* embryos were injected with the indicated mRNAs (0.8 ng Unc5BΔD, 0.16 ng Rnd1). Loss of adhesion was observed as ectodermal thinning and blastocoel edema at neurula stage 13 (upper row), as well as in paraffin sections of blastula stage 9 embryos (lower row). PPL, preprolactin (control mRNA). (D) Quantification of the deadhesion phenotypes from panel C (stage 9 embryos). (E) Rnd1 Morpholino (MO) injection rescues Unc5B-induced deadhesion. *X. laevis* embryos were injected sequentially with the indicated MO (40 ng) and mRNA (4 ng), and grown until blastula stage 9 in high-salt medium, which promotes ectodermal lesions when adhesion is inhibited (upper row) or in normal medium (lower row, paraffin sections). (F) Quantification of the Rnd1 MO rescue. Two embryo batches (exp.1,2) were injected as in panel E, grown in normal medium and cell deadhesion was assessed at blastula stage 9.

In coimmunoprecipitation assays Unc5BΔD (which causes deadhesion) interacted robustly with Rnd1, whereas Unc5BΔC (not causing deadhesion, see Figure 2A), as well as an unrelated receptor (BMPR1a) did not interact with Rnd1 (Figure 5A). The specificity of the Unc5B-Rnd1 interaction was corroborated by the lack of Unc5B binding to the related small GTPase RhoA (Figure 5B).

We next tested whether Unc5B and Rnd1 can functionally synergize. When low doses of Unc5BΔD and Rnd1 mRNAs were separately injected into *X. laevis* embryos, no significant deadhesion occurred, but their combined injection resulted in severe loss of embryonic cell adhesion (Figure 5C,D).

To examine whether Unc5B-mediated loss of cell adhesion requires Rnd1, as was demonstrated for FLRT3 [4], we injected Unc5BΔD mRNA in *X. laevis* embryos pre-injected with Rnd1 MO and monitored cell deadhesion at blastula stage (Figure 5E,F). The results showed substantial rescue of the deadhesion phenotype by Rnd1 MO. These data indicate an epistatic relationship where Rnd1 acts downstream of Unc5B to mediate its cell deadhesion activity.

## Discussion

In this study we discover a novel function of the Netrin receptor Unc5B to physically and functionally interact with FLRT3. We also find that Unc5B interacts with and signals via the small GTPase Rnd1 to inhibit cell adhesion. Taken together, the results from this and a previous study [4] suggest that Unc5B, FLRT3 and Rnd1 act together in regulating cell adhesion and morphogenesis in *Xenopus* development.

The LRR domains of FLRT3, which are essential for Unc5 binding, also mediate cell sorting of FLRT3 expressing cells [3]. Thus, FLRT3 appears to integrate two different activities: the LRR domains promote homotypic cell sorting/adhesion [3], while its intracellular tail confers cell deadhesion via Rnd1 [4]. During morphogenesis such bifunctionality may be crucial, because the same protein FLRT3 can both loosen adhesion in gastrulating cells, allowing them to move, and simultaneously keep homotypic cell groups together, preventing tissue disintegration. FLRT3 interactors, such as Unc5B, may modulate its overall adhesive properties.

Modulation of cell adhesion plays a key role in development, allowing complex coordinated tissue movements and cell rearrangements [19]. FLRT3 was proposed as an important player in *Xenopus* gastrulation, acting to inhibit cadherin adhesion through Rnd1 [4]. We extend these findings by implicating Unc5B in this process and showing that the Unc5B-FLRT3 interaction plays a role in *Xenopus* development. The loss of adhesion observed upon overexpression and the Morpholinos-induced defects in morphogenesis are consistent with a role of Unc5B-FLRT3 in convergent extension movements, which critically depend on cell adhesion [19], [20]. In early mouse development FLRT3 is also implicated in cell adhesion regulation and its ablation leads to various morphogenesis defects [5], [6]. It will be interesting to test whether Unc5 and Rnd proteins cofunction with FLRT3 in this context.

The mechanism of Unc5B-induced loss of adhesion is not clear at present. One possibility is C-cadherin internalization from the cell surface via endocytosis, as suggested for FLRT3 [4]. While our C-cadherin rescue experiment is consistent with the notion that cadherin adhesion is impaired, no significant changes of either total or cell surface levels of C-cadherin upon Unc5B or FLRT3 overexpression were detected. This may be due to technical limitations or experimental conditions, but may also indicate a different mechanism. Cadherin adhesion is subject to complex regulation at multiple levels from membrane clustering to cytoskeletal interaction [21]. Rnd proteins are strongly implicated as modulators of the actin cytoskeleton dynamics in cultured cells [22]. It will be therefore interesting to study whether FLRT3, Unc5B and Rnd1 affect cell adhesion in *Xenopus* embryos through the cytoskeleton. Notably, in cultured cells Rnd1 colocalises with cadherins in adherens junctions [23], raising the possibility that it regulates the interaction between the cadherin complexes and the cytoskeleton.

Unc5 proteins are best known as axon guidance receptors [9], [10]. In this regard it is noteworthy that FLRT and Rnd proteins have been individually implicated in neurite outgrowth regulation [7], [8], [22], [24]. Additionally, Unc5 receptors are involved also in vasculogenesis and apoptosis.[11], [12], [13], [14]. It therefore appears promising to test if FLRT and Rnd proteins collaborate with Unc5 in the above mentioned biological processes and whether FLRT interaction with Unc5 occurs in *cis* (within one cell) or in *trans* (between neighbouring cells). Ultimately, the here reported identification of novel membrane and intracellular Unc5 interactors may help elucidating how this important class of receptors exerts its diverse cellular functions.

## Methods

### Cell surface binding assays

Cells plated in poly-L-lysine-treated (Sigma, 100 µg/ml) 24-well culture plates were transiently transfected with expression constructs using FuGENE 6 (Roche). After 48 h, concentrated AP-FLRT3ΔTM conditioned media (3 to 8 U/ml AP activity) in AP-binding buffer (DMEM, 10% FCS, 50 mM HEPES) was added to the cells. After 1 h incubation at RT, cells were washed three times with PBS and stained for bound AP with Fast Red substrate (Roche). For binding curves, different amounts of concentrated AP or AP-FLRT3ΔTM CM in AP-binding buffer were added to transfected cells in 48-well plates (always at least in triplicates) and incubated for 1 h at RT. Cells were then harvested by pipetting, counted, washed 4 times with PBS containing 0.2 M NaCl, lysed in 70 µl per well 1% Triton X-100, 10 mM Tris (pH 8.0) and cleared by centrifugation. The supernatants were heated at 65°C for 1 h to inactivate endogenous cellular phosphatase activity. Finally, AP activity was determined by adding equal volumes of AP-substrate buffer (2 M diethanolamine pH 9.8, 1 mM MgCl_2_, 6.7 mg/ml Sigma 104 phosphatase substrate) to 20 µl cell lysates in 96-well microtiter plates and measuring the absorbance at 405 nm in an ELISA reader (Multiskan RC, Labsystems). Background counts were subtracted from each data point and the absorbance normalized to cell number was calculated.

### Embryological assays

*Xenopus* embryo culture and manipulations were carried out following standard protocols and details are described in the Supplement (Text S1). Morpholino (MO) antisense oligonucleotides (Gene Tools) were as following: standard Control MO, cctcttacctcagttacaatttata (Gene Tools); Rnd1 MO, tgggttccttcgttccttcatggtg [4]; FLRT3-ATG MO, attccaagtttctgtagacatggtc [4]; FLRT3-UTR MO, gtgtttccaagaatagagaggtctg; Unc5B-ATG MO, ggataaatgcatcgcttagcgtctc; Unc5B-UTR MO, acaatgccaaggtgtcccgcaagca. FLRT3-ATG and Unc5B-UTR MOs have one single terminal mismatch each in *X. tropicalis*, which should not significantly affect their activity [18].

Additional experimental methods and reagents are described in the Supplement (Text S1).

## Supporting Information

Figure S1 Spatio-temporal expression patterns of FLRT3 and Unc5B in Xenopus tropicalis development
(0.35 MB PDF)

Text S1 Supplementary Materials and Methods
(0.03 MB PDF)
