# Supplementary material for: Unc5B Interacts with FLRT3 and Rnd1 to Modulate Cell Adhesion in Xenopus Embryos
Source: PLoS One. 2009 May 29;4(5):e5742. doi: 10.1371/journal.pone.0005742 (PMC2683942; doi:10.1371/journal.pone.0005742)
Supplement: Text S1 — Supplementary Materials and Methods (0.03 MB PDF) [file pone.0005742.s002.pdf]

Supplementary Materials and Methods

Unc5B interacts with FLRT3 and Rnd1 to modulate cell adhesion in *Xenopus* embryos

Emil Karaulanov, Ralph T. Böttcher, Peter Stannek, Wei Wu, Marlene Rau, Souichi Ogata, Ken W.Y. Cho and Christof Niehrs

Expression Constructs. A mouse Unc5D cDNA in the pCMV-SPORT2 vector was isolated by expression cloning through a cell surface binding screen essentially as described [1]. A full-length *Xenopus laevis* Unc5B cDNA was cloned from a cDNA library through a PCR-based screen into the pCS2+ expression vector [2,3]. The corresponding GenBank accession numbers are FJ824810 and FJ824811. The open reading frames without the signal peptides of Unc5D (amino acids 32-938) and Unc5B (amino acids 36-942) were subcloned by PCR into pCS-V5 or pCS-FLAG vectors, which contain the mouse Kremen2 signal peptide (amino acids 1-26) followed by V5- or FLAG-tag sequences to generate N-terminal tags and deletion constructs [1,4]. Unc5D deletions are: ΔIG (Δ amino acids 1-238), ΔTSP (Δ amino acids 252-370), ΔTM (Δ amino acids 378-938). Unc5B deletions (in pCS-V5) are: ΔD (Δ amino acids 846-942) and ΔC (Δ amino acids 407-942). To generate AP-tagged *Xenopus laevis* FLRT3ΔTM, its extracellular domain without the signal peptide (amino acids 29-525) was cloned by PCR into pAPtag-4 (GenHunter) in frame with secreted human placental AP. The Renilla fusion constructs were made by PCR cloning [4]. Zebrafish Robo2 and -3 expression constructs were generated by subcloning the respective open reading frames [5] into pCS2+. V5-tagged BMPR1a was created by PCR cloning of *Xenopus* BMPR1a [6] into pCS-V5. Other constructs used for cell transfections and mRNA synthesis were: V5- and FLAG-tagged *Xenopus* FLRT3ΔTM in pCS-V5 and pCS-FLAG [4], FLAG-tagged *Xenopus* Rspo2ΔC in pCS-FLAG [7], FLAG-tagged *Xenopus* Dkk1 and mouse Dkk3 in pCS2+ [8], 3xHA-tagged *Xenopus* Rnd1 and RhoA in pCS2+ [9], full-length *Xenopus* C-cadherin in pcDNA3.1 [9], and PPL in pCS2mt [10]. Expression of all tagged constructs was confirmed by AP activity and/or by SDS-PAGE and immunoblotting.

Cell culture. HEK293T cells were cultured and transfected essentially as described [11]. Conditioned media (CM) for CoIP were produced by incubating transfected cells for 3 days in growth medium with 1% FCS. AP and AP-xFLRT3ΔTM CM were prepared with Opti-MEM I serum-free medium (Invitrogen) and concentrated using Centricon Plus-20 columns (Millipore).

Coimmunoprecipitation (CoIP) and immunoblotting (IB). For CoIP from CM, 0.5 ml of V5-FLRT3ΔTM CM was mixed with 1 ml of different FLAG-tagged CM with similar levels of protein expression. The samples were supplemented with 10% FCS and 20 mM HEPES and incubated for 6 h at

37 °C. IP was performed with 20 µl FLAG M2 agarose beads (Sigma) overnight at 4 °C with rotation. The beads were washed 4 times on ice with cell growth medium supplemented with 20 mM HEPES and 0.5% NP-40 detergent. After elution with gel loading buffer, the samples were analysed on 10% SDS-PAGE followed by V5 or FLAG IB. CoIP with Renilla fusion proteins was performed as described [11]. For CoIP from lysates, cells were cotransfected in 6-well plates with V5- and HA-tagged constructs and one day later lysed in 0.6 ml IP buffer (1% Triton X-100, 150 mM NaCl, 50 mM Tris pH 7.0, 0.1% BSA, protease and phosphatase inhibitors). After clearing by centrifugation, the lysates were incubated consecutively with 1 µl V5 antibody (Invitrogen) for 3 h and then with 20 µl Protein A-Agarose Fast Flow beads (Sigma) overnight at 4 °C with rotation. After 4 washes with IP buffer on ice, the beads were eluted in gel loading buffer and analysed on 10% SDS-PAGE followed by V5 or HA IB. Primary antibodies for IB were: anti-FLAG M2 (Sigma), anti-V5 (Invitrogen), anti-HA (clone 3F10, Roche), anti-C-cadherin (clone 6B6 [12], Developmental Studies Hybridoma Bank at the University of Iowa).

***Xenopus* assays.** Frog husbandry, *in vitro* fertilization, microinjection, embryo culture and staging, paraffin embedding and sectioning, RNA synthesis, *in situ* hybridization and pigment bleaching were carried out following standard protocols [10,13](http://tropicalis.berkeley.edu/home/). MO antisense oligonucleotides were designed according to the manufacturer's instructions and the available mRNA, EST and genomic sequence information. All mRNAs were injected 4 times into the animal region of 2-4 cell stage *X. laevis* embryos. The total dose of injected mRNA was kept constant by addition of preprolactin (PPL) control mRNA. After injection, embryos were cultured in normal medium (0.1x Barth) or alternatively in high-salt medium (1x Barth), which allows observation of ectodermal lesions upon inhibition of cell adhesion [14]. Deadhesion at blastula stage was also evaluated in freshly excised animal caps or in bisected embryos after fixation in 3.7% formaldehyde in isotonic buffer. The deadhesion phenotype was classified as strong (++) when most of the ectodermal cells were detached and weak (+) when most of them were still adherent. Paraffin sections of representative embryos were made to exemplify the observed loss of adhesion. Deadhesion was also quantified at neurula stage, where prior defects in ectodermal adhesion led to a characteristic, externally visible phenotype. Rnd1 MO was injected in *X. laevis* animally at 1-cell stage, followed by mRNA injection animally at 2-4-cell stage. FLRT3 and Unc5B MOs were injected in *X. tropicalis* equatorially at 1-2-cell stage and the total MO dose (40 ng per embryo) was kept constant in all samples by addition of standard Control MO (Gene Tools). Injected embryos were collected for marker analysis at gastrula and neurula stages, and also for *in situ* hybridization at gastrula stage; the remaining sibling embryos were evaluated for phenotypic defects at tailbud stage. For quantitative RT-PCR, a total of 10 embryos per sample were collected and RNA isolated with the RNeasy Mini kit (Qiagen). Following reverse transcription, quantitative RT-PCR were performed on a LightCycler 480 (Roche) in duplicates, the data was averaged, normalized to the housekeeping gene *ODC* and plotted on a linear scale.

Primers and probes for quantitative intron-spanning RT-PCR assays on a LightCycler 480 (Roche)

Gene	Forward primer	Reverse primer	UPL probe
ODC	tttggtgccacccttaaaac	ccactgccaacatggaaac	#50
FLRT3	aatgaagtgggtgcgtgact	agcccacgtacgttaactttg	#13
Unc5A	ggactgtgcgcctaaagaag	tagtggtgtgatgggcttcc	#33
Unc5B	tcaaggcaactgggagga	cccagctggtcaagcaaa	#51
Unc5C	gaagttttgctgcaatgtcg	ctcatttttcagccattccac	#17
Unc5D	agtcaatcaaggggaaccaa	tcaggtcccagaagaacctc	#12
Xbra	ttcaaggagctcaccaatga	cgacacgctcacctttagaa	#97
Xnot	acaacagcagccaatgagg	ggcaatgggagtagggtaaac	#96
Chordin	aggagcccctccaatctaag	gatgccatgaatcctccaga	#31
Dkk1	caaataccaacctttggatgc	tctggaactgtggcagaactc	#37
Bambi	ctttaaatggatcgccaagc	gagcgtcacagtagcatctga	#106
Vent2	ggcttctgagagacggaaac	ttgtatttcatcctgcggttc	#78
Gata2	ggtgttttacagccaagctca	ggctatgcaacaggtgagg	#36
MyoD	ggtccaactgctccgatg	ctgctgtcgtagctgttcctt	#1
Myf5	agctgctcagatggcatga	agctgctgttccttccagac	#66
Sox3	agccatcacctcccacac	caggtacatgctgatcatatctcg	#14
N-tubulin	taaccatgcgtgaaatcgtg	ggatcaattccgtgttcatca	#64
Slug	acatccgcactcacactgg	atcagaatgggtctgcaggt	#112
Snail	cctgcgtctgcaaaatctg	gcagtgcgtacaggaaaatg	#133
Epidermal Keratin	acggtcacttcagagcttgg	gaggttgccttcaactgattg	#33

References

1. Mao B, Wu W, Davidson G, Marhold J, Li M, et al. (2002) Kremen proteins are Dickkopf receptors that regulate Wnt/beta-catenin signalling. Nature 417: 664-667.
2. Rupp RA, Snider L, Weintraub H (1994) Xenopus embryos regulate the nuclear localization of XMyoD. Genes Dev 8: 1311-1323.

3. Turner DL, Weintraub H (1994) Expression of achaete-scute homolog 3 in Xenopus embryos converts ectodermal cells to a neural fate. Genes Dev 8: 1434-1447.
4. Böttcher RT, Pollet N, Delius H, Niehrs C (2004) The transmembrane protein XFLRT3 forms a complex with FGF receptors and promotes FGF signalling. Nat Cell Biol 6: 38-44.
5. Lee JS, Ray R, Chien CB (2001) Cloning and expression of three zebrafish roundabout homologs suggest roles in axon guidance and cell migration. Dev Dyn 221: 216-230.
6. Graff JM, Thies RS, Song JJ, Celeste AJ, Melton DA (1994) Studies with a Xenopus BMP receptor suggest that ventral mesoderm-inducing signals override dorsal signals in vivo. Cell 79: 169-179.
7. Kazanskaya O, Glinka A, del Barco Barrantes I, Stannek P, Niehrs C, et al. (2004) R-Spondin2 is a secreted activator of Wnt/beta-catenin signaling and is required for Xenopus myogenesis. Dev Cell 7: 525-534.
8. Mao B, Wu W, Li Y, Hoppe D, Stannek P, et al. (2001) LDL-receptor-related protein 6 is a receptor for Dickkopf proteins. Nature 411: 321-325.
9. Ogata S, Morokuma J, Hayata T, Kolle G, Niehrs C, et al. (2007) TGF-beta signaling-mediated morphogenesis: modulation of cell adhesion via cadherin endocytosis. Genes Dev 21: 1817-1831.
10. Gawantka V, Delius H, Hirschfeld K, Blumenstock C, Niehrs C (1995) Antagonizing the Spemann organizer: role of the homeobox gene Xvent-1. EMBO J 14: 6268-6279.
11. Karaulanov EE, Böttcher RT, Niehrs C (2006) A role for fibronectin-leucine-rich transmembrane cell-surface proteins in homotypic cell adhesion. EMBO Rep 7: 283-290.
12. Brieher WM, Gumbiner BM (1994) Regulation of C-cadherin function during activin induced morphogenesis of Xenopus animal caps. J Cell Biol 126: 519-527.
13. Sive HL, Grainger RM, Harland RM (2000) Early Development of *Xenopus laevis*: Cold Spring Harbor Laboratory Press.
14. Bisson N, Poitras L, Mikryukov A, Tremblay M, Moss T (2007) EphA4 signaling regulates blastomere adhesion in the Xenopus embryo by recruiting Pak1 to suppress Cdc42 function. Mol Biol Cell 18: 1030-1043.
