# Supplementary material for: Unc5B Interacts with FLRT3 and Rnd1 to Modulate Cell Adhesion in Xenopus Embryos
Source: PLoS One. 2009 May 29;4(5):e5742. doi: 10.1371/journal.pone.0005742 (PMC2683942; doi:10.1371/journal.pone.0005742)
Supplement: Figure S1 — Spatio-temporal expression patterns of FLRT3 and Unc5B in Xenopus tropicalis development (0.35 MB PDF) [file pone.0005742.s001.pdf]

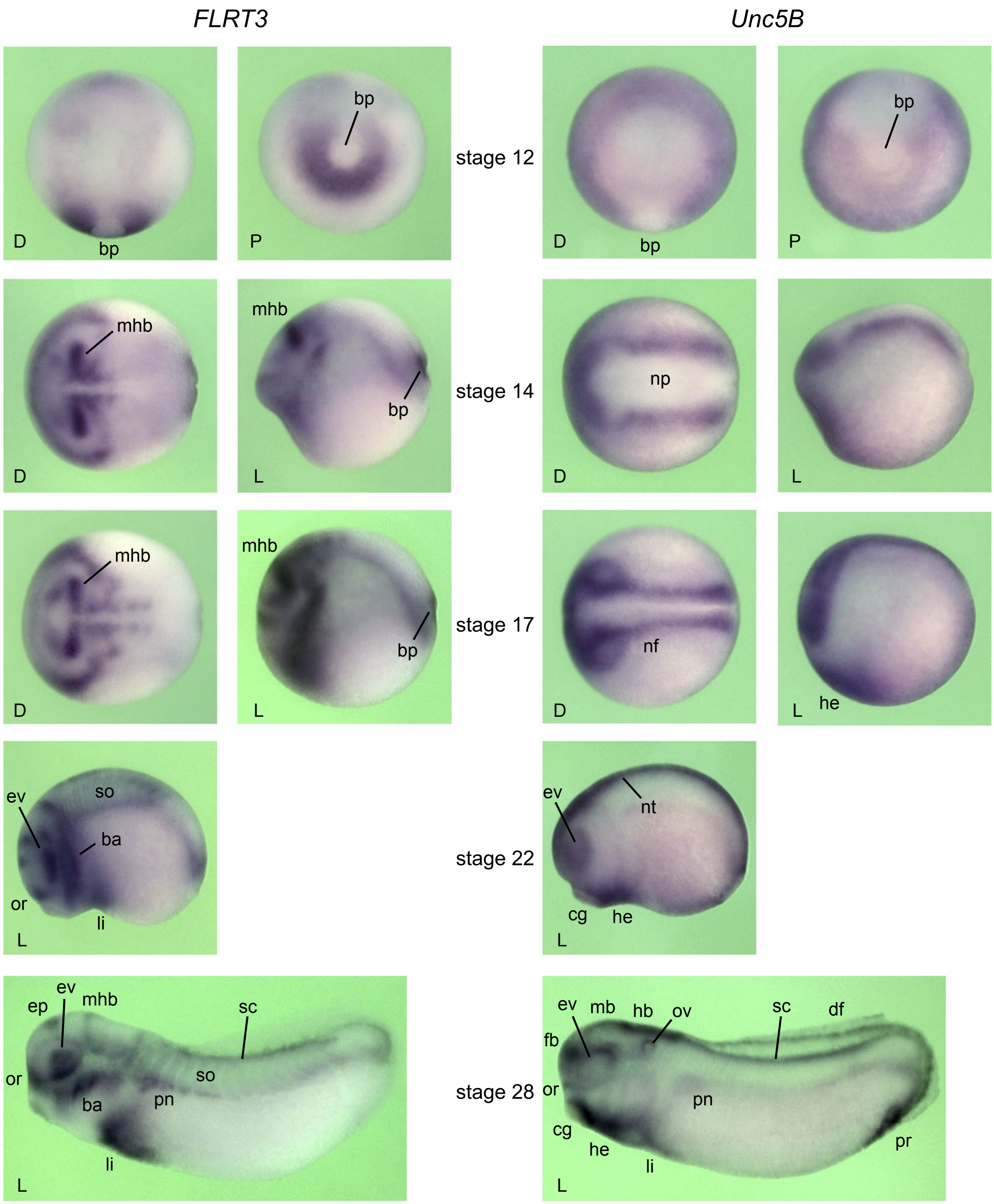

Figure S1. Whole mount in situ hybridisation of *FLRT3* and *Unc5B* in *Xenopus tropicalis* embryos at the indicated developmental stages. Views: D – dorsal, P – posterior, L – lateral. Embryonic structures and expression domains: bp – blastopore, mhb – mid-hindbrain boundary, np – neural plate, nf – neural fold, he – heart field, ev - eye vesicle, so – somites, ba – branchial arches, li – liver, or – oral region, nt – neural tube, cg - cement gland, ep – epiphysis, pn – pronephros, fb – forebrain, mb - midbrain, hb – hindbrain, ov – otic vesicle, sc - spinal cord, df –dorsal fin, pr – proctodeum.
